# Development of gold nanoparticle-based biosensors for COVID-19 diagnosis

**DOI:** 10.1186/s43088-022-00293-1

**Published:** 2022-09-05

**Authors:** Johra Khan, Yousef Rasmi, Kevser Kübra Kırboğa, Ahmad Ali, Mithun Rudrapal, Rohan R. Patekar

**Affiliations:** 1 Department of Medical Laboratory Sciences, College of Applied Medical Sciences, Majmaah University, Al Majmaah, 11952 Saudi Arabia; 2 Health and Basic Sciences Research Center, Majmaah University, Al Majmaah, 11952 Saudi Arabia; 3 Cellular and Molecular Research Center, Urmia University of Medical Sciences, Urmia, Iran; 4 Department of Biochemistry, School of Medicine, Urmia University of Medical Sciences, Urmia, Iran; 5 Bioengineering Department, Suleyman Demirel University, Isparta, 32260 Turkey; 6 Department of Life Sciences, University of Mumbai, Mumbai, 400032 India; 7 Department of Pharmaceutical Chemistry, Rasiklal M. Dhariwal Institute of Pharmaceutical Education and Research, Pune, Maharashtra 411019 India; 8 Department of Pharmaceutical Sciences and Natural Products, Central University of Punjab, Bathinda, Punjab 151401 India

**Keywords:** Quantum dot, Carbon nanotube, Gold nanoparticles, Point-of-care testing, SARS-CoV-2, COVID-19 diagnosis

## Abstract

**Background:**

Severe acute respiratory syndrome coronavirus 2 (SARS-CoV-2) is the causative organism of coronavirus disease 2019 (COVID-19) which poses a significant threat to public health worldwide. Though there are certain recommended drugs that can cure COVID-19, their therapeutic efficacy is limited. Therefore, the early and rapid detection without compromising the test accuracy is necessary in order to provide an appropriate treatment for the disease suppression.

**Main body:**

Nanoparticles (NPs) can closely mimic the virus and interact strongly with its proteins due to their morphological similarities. NPs have been widely applied in a variety of medical applications, including biosensing, drug delivery, antimicrobial treatment, and imaging. Recently, NPs-based biosensors have attracted great interest for their biological activities and specific sensing properties, which allows the detection of analytes such as nucleic acids (DNA or RNA), aptamers, and proteins in clinical samples. Further, the advances of nanotechnologies have enabled the development of miniaturized detection systems for point-of-care biosensors, a new strategy for detecting human viral diseases. Among the various NPs, the specific physicochemical properties of gold NPs (AuNPs) are being widely used in the field of clinical diagnostics. As a result, several AuNP-based colorimetric detection methods have been developed.

**Short conclusion:**

The purpose of this review is to provide an overview of the development of AuNPs-based biosensors by virtue of its powerful characteristics as a signal amplifier or enhancer that target pathogenic RNA viruses that provide a reliable and effective strategy for detecting of the existing or newly emerging SARS-CoV-2.

## Background

The novel severe acute respiratory syndrome coronavirus 2 (SARS-CoV-2) has undoubtedly created an emerging disease that is a public health priority worldwide [1–6]. This global pandemic has highlighted the urgency of accurate, rapid, and cost-effective diagnostic tests for epidemic understanding and management by monitoring the world’s population [7–10]. Recently, researchers have focused on developing rapid detection systems because the monitoring and managing of the pandemic are extremely critical. The most widely used current diagnostic method, real-time polymerase chain reaction (RT-PCR) testing, is the gold standard and the most widely available diagnostic tool for SARS-CoV-2 detection [11, 12]. In some countries, it is the only way to declare official results. Other methods are designed on the immunoglobulins detection such as immunoglobulin M (IgM) and/or immunoglobulin G (IgG) [13, 14]. The detection of virus-specific genes by single-stranded DNA probes is of particular interest because of their high sensitivity and specificity compared to antibody- or antigen-based immunological methods for the early diagnosis of viral infections. Many of these molecular and immunological tests have been validated by the Food and Drug Administration (FDA), and commercial kits have been introduced in the field [15].

The development of an efficient, inexpensive, and rapid detection kit will allow infected individuals to perform tests without special knowledge, saving time, and resources. In addition to clinical diagnostic techniques, point-of-care (POC) diagnostic systems can pave the way and start a new era for virus screening [16]. However, considering the specificity- and sensitivity-based shortages and the vulnerabilities in monitoring the spread of the virus, there is a great need to develop integrated intelligent devices based on novel, secure, fast, and accurate diagnostic techniques and implement them on a large scale to curb this outbreak in the world. [15]. Nanoparticles (NPs) are widely used in many medical applications, such as biosensing, drug delivery, imaging, and antimicrobial therapy. Of the nanoparticles, gold nanoparticles (AuNPs) are the most commonly used NPs for viral diagnostic detection due to their unique optical properties, stability, and biocompatibility properties. Over the past 20 years, the unique physicochemical properties of AuNPs have been significantly utilized in clinical diagnostics [17].

The surface plasmon resonance (SPR) phenomenon of AuNPs is responsible for their intense colors, and large absorption and cross-sectional scattering properties [18]. As a result, a number of colorimetric AuNPs-based detection methods have been developed [19–22]. As in many different technological sections, NPs have demonstrated their appropriateness for biosensing applications. Among noble metal NPs, AuNPs are mostly used for biosensor application [23] due to their biocompatibility, their optical and electronic properties, and their relatively simple production and modification [24]. Like many other technological sections, NPs are suitable for biosensing applications. Of the noble metal NPs, AuNPs are used primarily for biosensor applications [23] due to their biocompatibility, optical and electronic properties, and relatively simple fabrication and modification [24].

This review describes how AuNPs-based biosensors can be used for the detection of SARS-CoV-2 in clinical samples with an aim to develop it as a diagnostic test for COVID-19.

## Main text

### Overview of SARS-COV-2 diagnosis

A large-scale diagnosis of SARS-CoV-2 regulates the spread within and across communities and will help to reduce the new coronavirus pandemic [25]. Diagnosis is currently based on a multiplex of criteria, including epidemiology, clinical symptoms, and in vitro diagnostics [14]. Currently, COVID-19 is diagnosed primarily by direct detection of SARS-CoV-2-RNA by nucleic acid amplification assays, most commonly by RT-PCR from the upper respiratory tract [26, 27]. It is complemented by other additional tests, including serological and radiological tests [14, 28].

Various RT-PCR assays are used worldwide; various assays amplify and detect different regions of the SARS-CoV-2 genome. Some target two or more genes, including the nucleocapsid (N), envelope (E), and spike (S) genes, as well as the regions of the first open reading frame, including the RNA-dependent RNA polymerase (RdRp) gene [29]. Although the E assay is specific for all SARS-CoV-associated viruses, the RdRp test only detects COVID-19 virus, but for laboratory confirmation, it is recommended that E be followed by RdRp. However, in areas where SARS-CoV-2 is widely spread, positive RT-PCR test result needs detection of at least one target gene, with priority to the E gene being more sensitive [29]. Other, less common types of nucleic acid amplification assays include isothermal amplification, clustered regularly interspaced short palindromic repeats (CRISPR)-based assays, and next-generation sequencing [30–32]. Rapid RT-PCR assays provide similar performance comparably to standard laboratory-based nucleic acid amplification assays, but rapid isothermal assays are less sensitive [33, 34].

Serological tests detect antigens and antibodies directed against the coronavirus. SARS-CoV-2 belongs to the same β-coronavirus family that caused the severe acute respiratory syndrome (SARS) and Middle East respiratory syndrome respiratory syndrome (MERS) epidemics, and it is expected to have a similar antibody production process [35] where there is a delay of 14–28 days after the onset of the disease until the antibodies appear in the serum of patients [36]. In setting where access to nucleic acid amplification tests is limited or expensive, antigen tests can be used as an initial test, but antigen tests are less sensitive than nucleic acid amplification tests, and negative antigen tests should be confirmed by additional tests. Assays that detect SARS-CoV-2 antigen can be performed quickly and at the point of care and allowing faster access to results than some nucleic acid amplification assays. Antigen assays are generally less sensitive than nucleic acid amplification assays [37–39]. Nevertheless, they can be useful when nucleic acid amplification tests are not available or where nucleic acid amplification tests turnaround times are too long to be clinically useful, provided that clinicians are aware of the possibility of false negative results and interpret the results according to the pretest probability of COVID-19.

For more accurate results, a combined IgG and IgM antibody test is recommended [35]. Serologic tests detect antibodies to SARS-CoV-2 in the blood, and those that have been adequately validated can help identify patients who previously had SARS-CoV-2 infection as well as patients with current infection who have had symptoms for three to four weeks. Serological tests have very limited diagnostic benefit in acute cases because they are less likely to be reactive in the first days to weeks of infection [27, 40]. Because the sensitivity of the test is uncertain after 5 weeks, the accuracy of the test is optimized by confirming the serological test 3–4 weeks after the onset of symptoms [41]. In the reported study, the mean time to seroconversion was 12 days, while positive RT-PCR is observed 5–6 days after the onset of symptoms, suggesting that antibody testing is still worse than RT-PCR in COVID-19 diagnosis but more likely used when RT-PCR is not available or accessible [42].

### Nanotechnology-based biosensors

Use of biosensors is the most recent and advanced tool for diagnostics. The nanotechnology-based biosensors consist of a receptor (receive samples), a transducer (to processes sample), and a reading system to indicate result [43]. The successes of biosensors are due to their high sensitivity and specificity, whereas the use of nanomaterials makes them able to react to biomarkers at low potential [44]. Continuous monitoring of patient health status and a rapid decision making are two vital steps for better health care services during pandemic situation like COVID-19 [45].

The recent developments in nanotechnology made it possible to use different nanomaterials as electrodes in nanosensors resulting in production of nanobiosensors. Various nanomaterials such as nanofilms, nanowires, nanotubes, and nanotubes of different materials are either under use or development stages. Some of these nanomaterial-based biosensors are described as follows:

#### Quantum dots-based biosensors

Quantum dot (QD)-based nanoparticles range from 2 to 10 nm in size with unique electrical and optical properties and a very versatile nature [46]. These particles are made of cadmium selenide or indium phosphate core, an outer shell of zinc sulfide, and an upper most organic coating to give a hydrophilic property to the particle for its better connectivity with biomolecules (proteins and oligonucleotides) [47]. Due to broad spectrum property of QD-based nanoparticles from red to blue light make them suitable  to be used in medical imaging, labeling, and sensing biosensors specially to differentiate between normal and tumor cells [48]. Some researchers used magnetic QD and magnetic nanoparticles for antibiotic detection. Meng and co-workers developed an innovative method to identify five antibiotic residues (quinolone) in various food items [49]. Li et al. combined QDs with Eu^3+^ for detecting tetracycline in environmental and biological samples [50].

#### Carbon nanoparticle- and nanotube-based biosensors

Use of carbon nanoparticles and nanotubes (CNT) in biosensors is introduced due to their low cost [51], well-organized structure, and having combination of many unique properties including magnetic, electric, and chemical ones [52]. CNT are important due to their capacity to pass biological barriers easily and can penetrate individual cells easily [53]. Carbon nanoparticle-based and CNT-based biosensors are used for various diagnostic methods including enzymes, antibody, polypeptides, DNA, RNA, and aptamer. Lubbers and Oppitz developed first fiber optics-based biosensor known as ‘optode’ to measure CO_2_, O_2_, and alcohol in cells and tissues [54]. Nanobiosensors based on CNT are used to detect DNA sequences related to specific disease condition. These biosensors can be easily inserted in body without causing inflammation and can eliminate need of blood drawing or repeated sample collection.

#### Metal nanoparticle-based biosensors

AuNPs are commonly used for the development of electrochemical and optical biosensors [55]. Wei et al. constructed AuNP biosensors with boron-doped diamond electrodes to detect organophosphate pesticide, which is used in agriculture. AuNPs on carbon spheres are used as colorimetric biosensors for detection of oligonucleotide or DNA [56]. AuNP-based biosensors are sensitive to 10^–11^ mol/L. Due to its high sensitivity, Su et al. [57] used AuNPs-based biosensors for *Bacillus anthracis* using QCM (quartz crystal microbalance). They immobilized thiolated DNA probe on AuNP having a complementary DNA sequence with extended sequence. After introduction, the signals amplified with DNA-conjugated AuNP lower the detection limits of bacteria by 3.5 × 10^2^ CFU/ml and reduce the use of carcinogenic reagents for detection [57]. The recent researches on nanomaterial-based biosensors are still going on with many modifications, and area of diagnostics is increasing as in case of COVID-19. AuNP-based biosensors are in use and development for the diagnostic use in detecting and monitoring COVID-19 disease.

### Gold nanoparticle-based biosensors

**Fig. 1:**
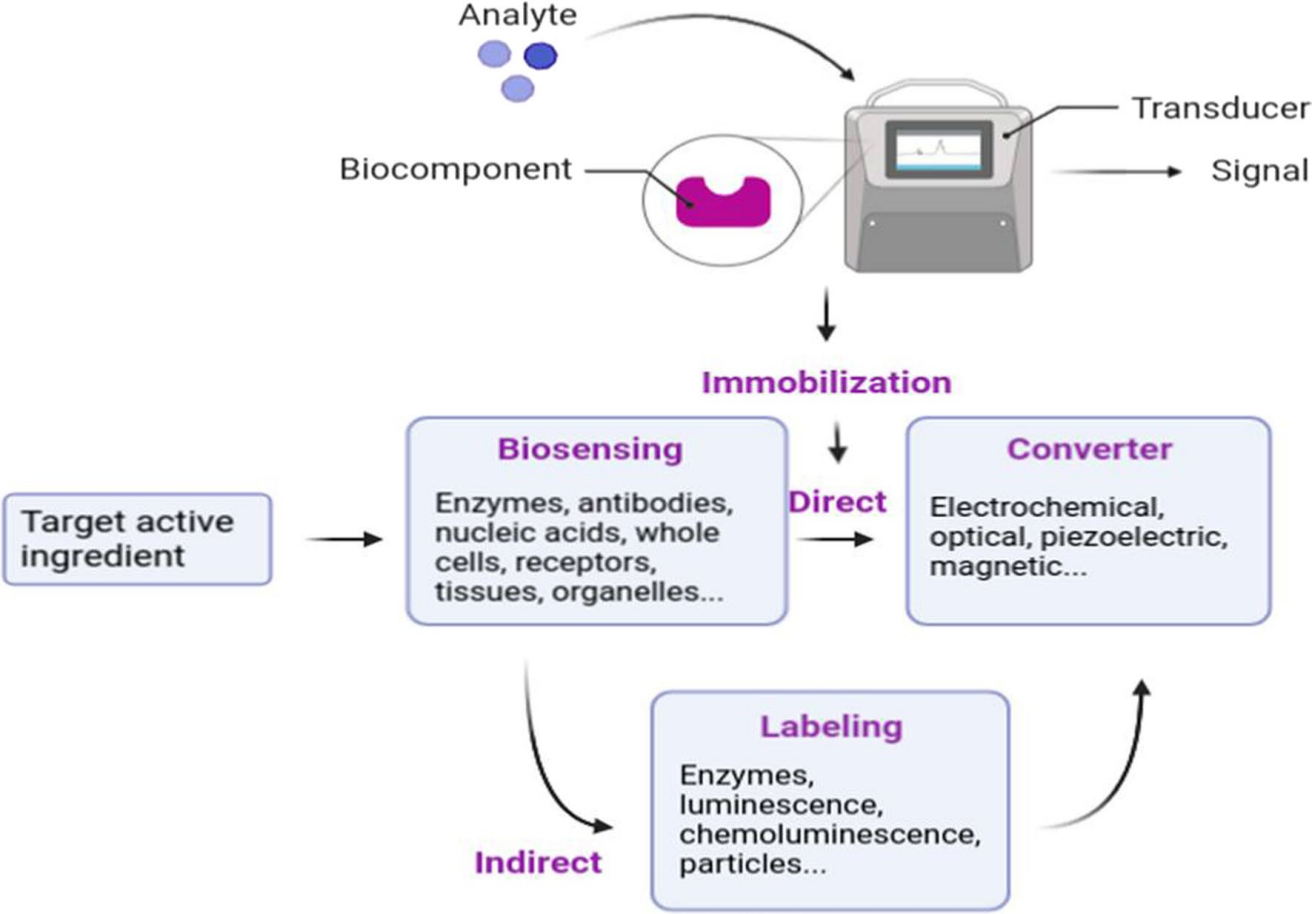
Typical structure and components of a biosensor

AuNPs have the characteristics of being inert, biocompatible, and non-toxic, as well as being easy to synthesize, changing their surface functionality, and being adjustable in size and shape. Therefore, it has stimulated interest in interfacing biorecognition systems with signal transduction and as determining agents in the design and implementation of functional biosensing devices [58, 59]. Biosensors that generate signals in proportion to the amount of analyte to be analyzed have many uses. These are mainly viral diagnosis during the COVID-19 pandemic period, other clinical diagnoses, monitoring of clinical processes, bioreactors, drug production and release. Achieving results in a short time and ease of application are the most important advantages of biosensors [60] (Fig. 1). AuNPs-based biosensors can be classified as optical biosensors, electrochemical biosensors, and piezoelectric biosensors. Below is an introduction to various types of GNP-based biosensors. The focus is on the effects of AuNPs on the biosensing process and the mechanism of improving analytical performances (Table 1).

**Table 1:** Different functions of AuNPs in biosensors

Types of biosensors	Principle of function	Functions of AuNPs	Sensor advantages
Optical biosensors	It works with the principle of creating a light beam with certain characteristics from the light source	Improve the process of change of refractive index	Developed sensitivity
Electrochemical biosensor	Based on utilization/production of electrons and reaction of enzymatic catalysis	Catalysis of reaction	Developed sensitivity and selectivity
Piezoelectric biosensors	It is based on measuring the mass of the sample collected on a piezoelectric crystal surface by detecting the difference in resonance frequency	Effect on enzyme immobilization and mass changes	Developed sensitivity

#### Development of gold nanoparticle-based biosensors

With the development of AuNPs-based biosensors, the properties of AuNPs have been used more extensively in the diagnostic process. The most critical point during development and design is that system behaviors are predictable and repeatable. Nano-PCR, an important tool for the diagnosis of viral diseases, is highly sensitive and dependent on the effects of AuNPs. AuNPs can be easily functionalized according to their shape, size, and aggregation properties. Hamdy et al. [61] reported that a new AuNPs biosensor was developed, using oligonucleotides recognizing certain genes of FMDV by developing a specific technique to diagnose the foot-and-mouth disease virus [61, 62]. The development of AuNPs-based biosensors based on their interaction with different substrates has also been very useful in colorimetric studies. Biosensors need to be modified and improved for long-term reliability, extended shelf life, and customization of operation and design [63].

Particularly for SARS-CoV-2, which is the COVID-19 virus, an economical and rapid test methodology has been developed to combat virus-induced pandemics by detecting nucleocapsid (N) proteins and for POC (point-of-care) applications. The methodology was combined with the localized plasmon resonance (LPR) principle of collecting antigen-coated AuNPs, and quantitative concentration values were measured with optical spectrometers. In this way, the presence of viruses can be detected by color change [32].

As mentioned above, AuNPs have excellent photoelectric properties and also resist DNAzyme transfection and nuclease degradation. For this reason, DNAzyme–AuNPs complexes have been the focus of attention in biosensing and bioimaging. It is used as a probe to increase and maintain catalytic efficiency, detect the molecular recognition signal, and convert it into a detectable physicochemical signal. In the future, these probes are very important for the detection of targets [62]. The development of versatile biosensors requires modulation and coordinated operation of different components. Although efficient synergy is not always possible, building a multifunctional biosensor will effectively reduce costs. In a related study, a three-dimensional cluster of AuNPs/ferrocene/liposome was fabricated to fabricate the electrochemical biosensor. In this way, electrochemical analysis of lipopolysaccharides (LPS) has shown great potential in terms of significant versatility and economic cost [63]. Another developed biosensor system is on viruses. They developed a biosensor based on displacement amplification using magnetic beads and enzymes in order to detect the pathogen in a shorter time and with less cost for the hemorrhagic fever virus, which is the pathogen of viral hemorrhagic fever disease (VHF) [54]. Hepatitis B virus (HBV), which causes cirrhosis, liver cancer, and many health problems if not managed properly, needs a precise diagnostic technique. For this purpose, various biosensors have been developed and allow timely intervention against this virus [64, 65]. Ultra-sensitivity and small size are important requirements in the development of new biosensors. Various microfluidic technologies integrated with technologies such as nanotubes, nanoparticles, detectors, and biosensors will be able to detect biological and chemical active substances faster, more precisely, at a lower cost, and more sensitively in many areas [66].

In another study with nanomaterials, oxidized thin films and AuNPs were combined and the capacitance values between DNA immobilization and hybridization were measured by means of an electrochemical DNA biosensor, and it was seen that AuNPs and thin films were successful in DNA detection [67].

#### Evolution of gold nanoparticle-based biosensors

The optical, chemical, and physical properties of AuNPs make them strong candidates for designing new biosensors and imaging methods. The development of AuNPs-based biosensors offers cost-effective and rapid strategies in unexpected conditions such as the COVID-19 pandemic process. The promising analytical behavior and unique and superior properties of AuNPs have shown advantageous performances for three main types of biosensors. Most diagnoses in the COVID-19 pandemic continue to be made by PCR, a costly and lengthy process. It is clear that virus-induced pandemics can be overcome with the development of AuNPs-based biosensors. Biosensors need agents with properties such as reliability, selectivity, and sensitivity in order to perform more analytically. Although there have been many developments in AuNPs-based biosensors, a widespread practical use is currently not available. Some challenges must be overcome in order for these biosensors to reach their full potential. But the generation of long-term AuNPs-based biosensors is promising. The use of AuNPs in biosensors should be encouraged, as their optical and electrical properties greatly affect the biosensors. As exemplified above, combining it with other nanomaterials and creating a synergistic effect remain interesting in the diagnostic process. In addition to the discovery of these new structure processes, preventing the adsorption process, obtaining reproducible results, and shortening the analysis time will improve the analytical performance of biosensors [68–71].

## Gold nanoparticle-based biosensors in COVID-19 diagnosis

To confirm SARS-CoV-2 infection, diagnostic procedures that identify viral nucleic acids, viral antigens, or serological testing are necessary. Rapid antigenic and rapid antibody tests have shorter execution times (15–30 min), a cheaper cost, and a simpler method that really does not need highly skilled staff [72]. The majority of COVID-19 immunoenzymatic serological tests are based on the indirect enzyme-linked immunosorbent assay principle (ELISA) [73]. This method allows researchers to acquire extremely precise and sensitive data in a short period of time (between 1 and 3 h). There are a variety of alternative methods for diagnosing COVID-19 infection, such as viral culture and electron microscopy, NGS, clinical investigations and imaging techniques (CT Scan), biosensor COVID-19 testing techniques, loop-mediated isothermal amplification (LAMP), CRISPR/Cas-based COVID-19 testing methods, and Digital PCR COVID-19 testing methods, but the effectiveness and specificity of these methods are still questionable [74].

The fast immunochromatographic test strip, also known as lateral flow immunoassay (LFIA), was created as a consequence of the confluence of numerous threads dating back to the era [75]. The notion of fast diagnostic tests based on bodily fluids, on the other hand, dates back far longer. The basic concepts of lateral flow technology were developed throughout the early 1980s and found throughout the latter years of that era, with businesses such as Becton Dickinson & Co., Unilever, and Carter Wallace submitting numerous important patents on this technological format [76]. The human pregnancy test, which reflected a continuing historical interest in urine testing for medical diagnostic purposes, was the dominant material driving the initial stages of solid-phase, rapid test technology [77]. Improvements in antibody production methods and substantial advancements in knowledge of the biology and detection of human chorionic gonadotropin (hCG), primarily due to Vaitukaitis and co-workers' work, propelled this specific diagnostic application forward in the 1970s. However, a variety of additional enabling technologies were necessary to properly create the lateral flow test platform [78].

### LFIA

Assays are traditionally made up of a range of components, each of which serves one or more roles. The components are layered on top of each other and adhered to a base card with pressure-sensitive epoxy. The assay consists of several zones including sample pad, conjugate pad, test line, etc. The sample is placed on the sample pad initially to make it compatible with the subsequent zones. This sample runs in the conjugate pad, which is the following zone. To produce a color response, the conjugate pad includes a small number of proteins, either antibodies or antigens, complexes with any enzyme or nanoparticle [79]. These proteins bind to the biological component in the sample that is present. These conjugate protein and biological sample complexes move to a new zone known as the test line [80]. This conjugate compound is caught by particular proteins in the test line. The conjugate enzyme is activated by interaction at the test line, resulting in a color response. To avoid cross-reactivity, the leftover proteins or biological components will be sent to the absorbent.

Improving test sensitivity and selectivity is critical for effective therapy. The existence of viral material and the measurement of the proportion of infection produced in the body are both significant. Scientists are working on new ways to improve the diagnostic selectivity and sensitivity. In the realm of nucleic acid detection, gold nanoparticles (AuNPs) have emerged as league nanomaterials [81]. The antigen–antibody complex is extremely simple during LFI, whereas in the case of AuNPs, antigen–antibody combination creates an aggregation with AuNPs. The AuNPs bind to multiple anti-SARS-CoV-2 antibodies and form a complex [82]. This complex when interacts with the SARS-CoV-2 antigens produces highly intensive color, which gives accurate results in colorimetry with good selectivity and specificity [83].

**Fig. 2:**
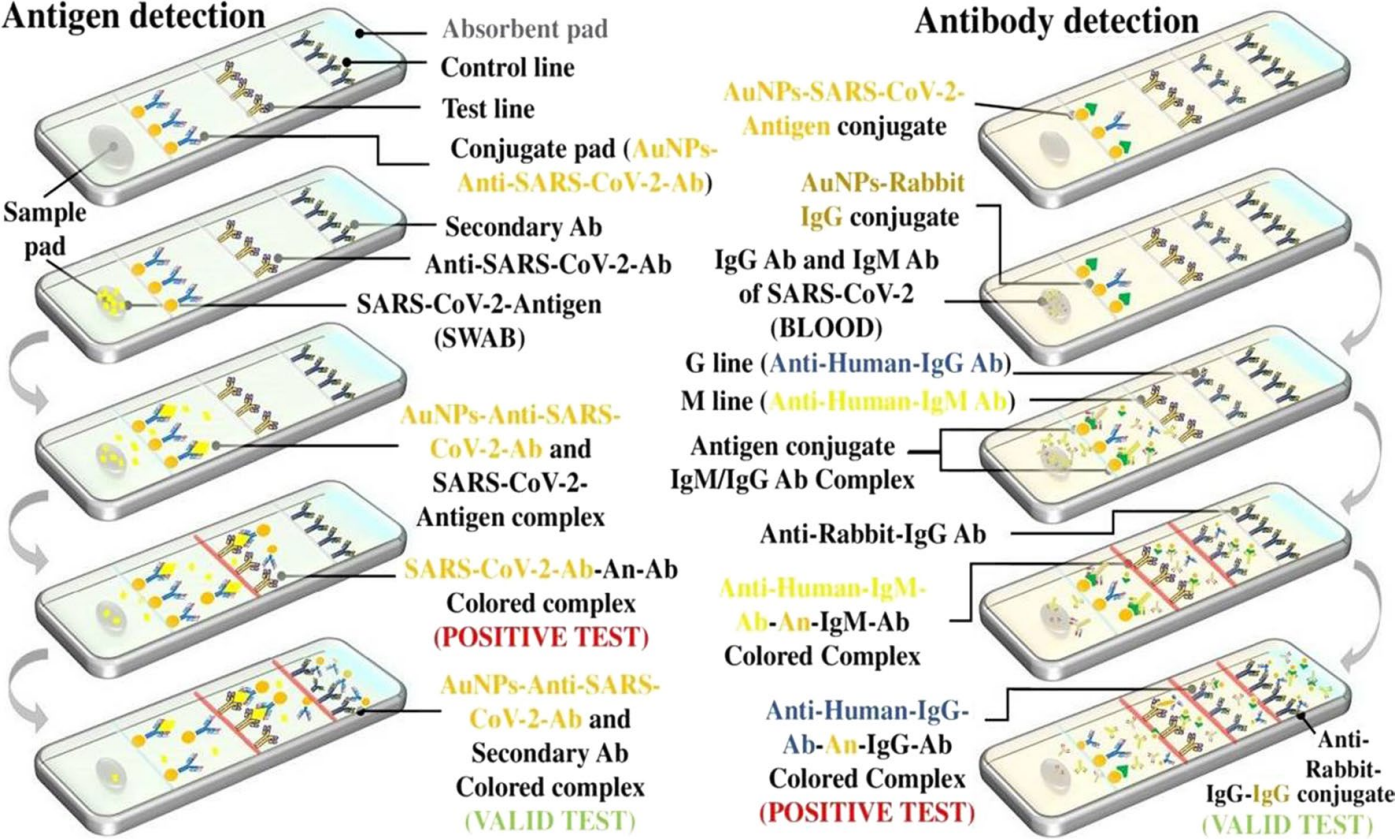
AuNPs in COVID-19 diagnosis

The gold nanoparticle diagnostic kit makes antigen identification from a swab simple. The Au anti-SARS-2 antibodies are included in the conjugate pad [84]. If the sample pad contains SARS-2 antigens, the antigens bind to the Au anti-SARS-2 antibodies throughout the run, forming antigen–antibody complexes. This binding is very complex because one gold nanoparticle binds to more than two anti-SARS-2 antibodies, indirectly causing multiple antigens to bind to one gold nanoparticle, resulting in a vibrant color test line. This vibrant color aids in accurate detection. Few Au anti-SARS-2 antibodies remain unbonded in the conjugate pad. The complexes of Au anti-SARS-2 antibodies and SARS-2 antigens, as well as unbound Au anti-SARS-2 antibodies, will rush to the test line [85]. The Au anti-SARS-2 antibodies–SARS-2 antigens complexes bind to the anti-SARS-2 antibodies at the test line, leaving the unbonded Au anti-SARS-2 antibodies unbound. The complexity of Au anti-SARS-2 antibodies–SARS-2 antigens–anti-SARS-2 antibodies rises to a next level, resulting in a very intense color generation. Secondary antibodies in the control line simply attach to the unbound Au anti-SARS-2 antibodies and produce color. These diagnostic kits are extremely easy to identify. The presence of SARS-2 antigens is shown by the color of the test line. The presence of Au anti-SARS-2 antibodies is indicated by the color on the control line, indicating that the kit is functioning properly [86]. The absence of color at the control line indicates that the kit is not in functioning order. The presence of the antigen (positive result) is shown by the color of the test and control lines, whereas the absence of the antigen (negative result) is indicated by the color only at the control line (Fig. 2).

The gold nanoparticle diagnostic kit makes antibody identification simple. The Au SARS-COV-2 antigens and Au rabbit IgG conjugate are included in the conjugate pad. If the sample pad contains SARS-COV-2 antibodies, the antibodies bind to the Au SARS-COV-2 antigens throughout the run, forming antigen–antibody complexes. This binding is very complex because one gold nanoparticle binds to more than two SARS-COV-2 antigens, indirectly causing multiple antibodies to bind to one gold nanoparticle, resulting in extreme color test line [87]. This extreme color aids in accurate detection. Au Rabbit IgG Conjugate and few Au SARS-COV-2 antigens remain unbonded in the conjugate pad. The complexes of Au anti-SARS-2 antigens and SARS-2 antibodies, as well as unbound Au Rabbit IgG Conjugate and few Au SARS-COV-2 antigens, will rush to the test line. For detection of the antibodies, there are two test lines (M line and G line) [88]. The Au anti-SARS-2 antigens–SARS-2 IgM antibodies complexes bind to the Anti-Human IgM antibodies at the M test line, leaving the unbonded Au anti-SARS-2 antibodies and Au anti-SARS-2 antigens–SARS-2 IgG antibodies unbound [89]. The Au anti-SARS-2 antigens–SARS-2 IgG antibodies complexes bind to the Anti-Human IgG antibodies at the G test line. The complexity of Au anti-SARS-2 antigens–SARS-2 antibodies–Anti-Human antibodies rises to a next level, resulting in a very intense color generation. Anti-rabbit IgG antibodies in the control line simply bind to the unbound Au rabbit IgG Conjugate and produce color. The presence of SARS-2 antibodies is shown by the color of the test line. The presence of Au rabbit IgG Conjugate is indicated by the color on the control line, indicating that the kit is functioning properly. The presence of the antibodies (positive result) is shown by the color of the test and control lines [90].

## Challenges and perspectives

At present, the impact of SARS-COV-2 virus on the immune system has been explored thoroughly. The well-implemented nanosystems have made the treatment procedure easier through the development of diagnostic tools [91]. However, the major challenge faced in implementation of diagnostic procedures globally is the variations observed in the outcomes with respect to region, race, gender, and age. Thorough insights to documented cases of COVID-19 clearly indicate that the severity, as well as symptoms, of infection presents itself as a unique scenario in every patient [92]. Nanotechnology can speed the diagnostic processes in mass population. However, for above reasons and the detection of severity of organ or systemic damage, a more personalized approach is required for effective patient treatment. To a large extent, this can be achieved through integration of nanotechnology techniques with that of artificial intelligence with the help of smart bedside monitoring systems. Through this integration, we can not only facilitate the diagnosis of infectious agents but also design evidence-based planning strategies for a more personalized treatment regimen [93].

On a molecular level, a major challenge is to develop biosensing devices with improved sensitivity to detect low titer of viruses in the samples. Considering the multiple processing steps involved during the use of biosensing devices like RNA extraction, cDNA amplification, and signal transduction, the sensitivity of the procedure can be easily compromised [94]. This necessitates the need for the use of ultra-pure reagents during development of biosensing devices and well-qualified and trained individuals for carrying out the diagnosis. Additionally, compliance with these factors alone is obscured in the absence of centralized biosafety laboratories required for handling biohazardous virus samples. Overall, these steps enhance the economic burden on the centralized healthcare systems as well as the community. To overcome these shortcomings, CRISPR-based devices used in ‘Point of Care’ diagnosis are suitable alternatives. However, they require more congruous approach and primer designs for every target nucleic acids [95].

Another technique for COVID-19 diagnosis based on serological antigen detection and antibody response is the preferred and standard diagnostic procedure for infectious diseases. However, these protein and enzyme-based techniques are highly sensitive to external factors and testing reagents. Besides, the cross-reactivity of these proteins with other coronaviruses can lead to false-positive results. Specifically, it is reported that the S protein of SARS-CoV frequently binds to the SARS-CoV-2 antibody leading to false-positive outcome. Also, the challenges of screening mass population during epidemics are enhanced with reliance on serological tests because they only detect the antibodies after it reaches a suitable titer after incubation.

## Conclusions

Epidemic outbreaks are a challenging scenario for the healthcare systems. We have witnessed a profound impact of COVID-19 pandemic on the economic and social aspects of the society. As summarized in this article, the use of nanotechnology-aided diagnostic approaches can help in rapid detection of SARS-COV-2 virus. In turn, it may allow early treatment of infection and gradually eradicate the virus by implementing thorough screening programs. In order to accomplish this goal, it is necessary to develop a reliable and universal diagnostic method that is affordable and highly sensitive and allows screening of mass population. Several advances with respect to diagnostic approaches using biomarkers, biosensors, and functional detection systems have been suggested in the literature. However, even the most suitable techniques are accompanied with several challenges. We are required to overcome these challenges soon to avoid the intensity of infectious diseases and in future prevent them from becoming a global epidemic. So far, the efficacy, stability as well as safety of nanotechnology-based diagnostic approaches as compared to other techniques hold promise in accomplishment of our goals to combat SARS-COV-2 infection.

## Data Availability

Not applicable.

## Abbreviations

IgG: Immunoglobulin M
IgG: Immunoglobulin G
FDA: Food and Drug Administration
SARS-CoV-2: Severe acute respiratory syndrome coronavirus 2
COVID-19: Coronavirus diseases 2019
SARS: Severe acute respiratory syndrome
MERS: Middle East respiratory syndrome respiratory syndrome
RT-PCR: Reverse transcriptase-polymerase chain reaction
POC: Point-of-care
NPs: Nanoparticles
AuNPs: Gold nanoparticles
SPR: Surface plasmon resonance
RdRp: RNA-dependent RNA polymerase
CRISPR: Clustered regularly interspaced short palindromic repeats
QD: Quantum dot
CNT: Carbon nanotubes
QCM: Quartz crystal microbalance
ELISA: Enzyme-linked immunosorbent assay principle
LFA: Lateral flow immunoassay
